# Determining the Risk of Developing Rheumatic Heart Disease Following a Negative Screening Echocardiogram

**DOI:** 10.3389/fcvm.2021.632621

**Published:** 2021-02-12

**Authors:** Meghan Zimmerman, Amy Scheel, Alyssa DeWyer, Jane-Liz Nambogo, Isaac Omara Otim, Alison Tompsett, Joselyn Rwebembera, Emmy Okello, Craig Sable, Andrea Beaton

**Affiliations:** ^1^Children's Hospital at Dartmouth Hitchcock, Lebanon, PA, United States; ^2^Dartmouth College, Hanover, IN, United States; ^3^School of Medicine, Emory University, Atlanta, GA, United States; ^4^School of Medicine, Virginia Tech Carilion, Roanoke, VA, United States; ^5^Kampala CardioLab, Kampala, Uganda; ^6^Uganda Heart Institute, Kampala, Uganda; ^7^Children's National Hospital, Washington, DC, United States; ^8^Cincinnati Children's Hospital Medical Center, Cincinnati, OH, United States

**Keywords:** rheumatic heart disease, pediatrics, echocardiography, screening, global health

## Abstract

**Background:** Screening echocardiograms can detect early-stage rheumatic heart disease (RHD), offering a chance to limit progression. Implementation of screening programs is challenging and requires further research. This is the first large-scale study assessing the risk of RHD among previous screen-negative children.

**Methods:** This retrospective cohort study, conducted in Gulu, Uganda, performed school-based echo screening on children ages 5–18 years. Surveys were used to determine which children underwent initial screening 3–5 years prior. Age, gender, and disease severity were compared between cohorts. Relative risk (RR) of RHD was calculated for those with a prior screen-negative echo (exposed cohort) compared to those undergoing first screening (unexposed cohort).

**Results:** Echo screening was completed in 75,708 children; 226 were excluded, leaving 1,582 in the exposed cohort and 73,900 in the unexposed cohort. Prevalence of new RHD was 0.6% (10/1,582) and 1% (737/73,900), in the exposed and unexposed cohorts, respectively. The RR of RHD was 0.64 (95% CI 0.3–1.2, *p* = 0.15), a nearly 40% reduced risk of RHD in those with a prior negative echo. There was no difference in age or gender between RHD cohorts. All cases in the exposed cohort were borderline/mild; 2.6% of cases in the unexposed cohort had moderate/severe disease.

**Conclusion:** There was no statistical difference in RHD prevalence between previous screen-negative children and children with no prior echocardiogram, however, there was a trend toward decreased risk and severity. This information has important implications for the design of screening programs and the use of screening echocardiograms in endemic RHD regions.

## Introduction

Rheumatic Heart Disease (RHD) is a common cause of cardiovascular morbidity and mortality in children and young adults globally. In 2017, the worldwide prevalence of RHD was over 39 million, with 249,000 RHD-related deaths (1, 2). In low-resource settings, clinical RHD is typically diagnosed at late stages of disease, most commonly with heart failure, resulting in high rates of morbidity and mortality (3). Screening echocardiograms (echos) have the ability to detect RHD at earlier stages and thereby create an opportunity to prevent further advancement of disease (4–6). In addition to the many logistic and financial barriers that exist for implementing echo screening programs in endemic regions, several knowledge gaps remain that require attention before echo screening can be recommended as public policy.

One key question is whether or not echo screening and early detection of disease will improve outcomes. An ongoing randomized control trial is designed to answer that question by evaluating the impact of Penicillin prophylaxis on RHD progression over time (GOAL Trial, Clinicaltrials.gov NCT03346525). In addition to studying the utility of screening, the optimal frequency and timing of screening has not been clearly delineated. Knowing the optimal frequency of screening would simultaneously cut unnecessary cost and use of resources, while minimizing the risk of disease progression between screening echos. Prior studies have evaluated small cohorts of children with mild or no RHD to evaluate progression over time, and have shown variable results (7–9). Our study is the first to follow a large cohort of children in an RHD endemic region to determine the risk of progression from normal to abnormal, based on screening echos, over time. This study was designed to determine the risk of RHD following one prior negative screening echo, with the hypothesis being that children with a prior negative screening echo will have lower rates of RHD detection than those never screened before. RHD is caused by a combination of environmental, genetic, and host factors (10). While any child can, in theory, develop RHD at any time, we know that some children are at higher risk. Therefore, a population with a single normal echocardiogram likely has a lower risk than the general population of later developing RHD.

## Methods

### Study Design

This retrospective cohort study was conducted in Gulu District, Uganda, located in Northern Uganda, with known endemic rates of RHD. The study enrolled children, ages 5 to 18 years, who attended primary and secondary schools in Gulu District. Initial screening echos were performed between 2013 and 2015 on 8,009 children, as part of three separate studies looking at the utility of hand-held echo to detect RHD (11–13). A second independent echo screening period was performed in primary and secondary schools between June and Sept of 2018. This extensive school-based screening was performed in 75,708 children in 165 schools in both rural and urban areas where prior screening had occurred. The second screening was embedded in a larger study to identify children with RHD in the community.

During the second screening, a short 4-question interview was conducted by members of the research team with each child to determine prior participation in echo screening. When children self-reported a prior echo screening, they were asked for their name, age, and prior school and class. This data was used to ensure adequacy of recall. All children with positive screening echos in the schools subsequently underwent a complete confirmatory echocardiogram, analogous to the process in the initial screening studies. World Heart Federation (WHF) criteria were used to confirm the diagnosis of RHD, evaluate valve morphology, and determine the severity of disease (14). Children were excluded from the study if: (1) they had a known diagnosis of RHD, diagnosed prior to the first screening period (based on the National Ugandan RHD registry), (2) had a diagnosis of other heart disease, (3) were outside the target age range (5–18 years), or (4) failed to return for the confirmatory echocardiogram.

### Sample Size

Sample size was determined using standard estimations for cross-sectional and cohort studies (15). “Exposed” was defined as having a prior negative screening echo during the initial screening period, while “unexposed” was defined as having no prior screening echo. Using previously published data, we assumed a conservative baseline population prevalence of 2.0%. Using independent *t*-tests, a sample size of 2,316 would be required in each group to detect a difference of 50% (1% in the exposed population), with an alpha of 0.05 and power (1-B) of 0.8 (16). This goal sample size seemed feasible, as 8,009 children were initially screened in the 2013–2015 timeframe, and the planned volume of the second screening period was >70,000 in Gulu District.

### Statistical Analysis

Statistical analysis was performed using SPSS statistical analysis software. Age, gender, and details of the initial screening echo (school name and class) were collected by in-person interviews for those in the exposed cohort, and for all participants with a positive screening echo. Median and interquartile range (IQR) were calculated for age in the exposed cohort and in those with RHD in both the exposed and unexposed cohorts. Within the exposure group, RHD and non-RHD groups were compared. Age was compared by independent *t*-tests and gender was compared using chi-square analysis. RHD prevalence was calculated for both exposed and unexposed cohorts. The absolute risk difference and relative risk (RR) were determined with 95% confidence intervals (CI). Statistical significance was deduced when the 95% CI of a RR did not include one and *p* < 0.05.

### Ethics

This study was covered under an existing Institutional Review Board (IRB) through Mulago Hospital at Makerere University, as part of the National RHD Outreach Program (REC REF 2013-072).

## Results

**Figure 1:**
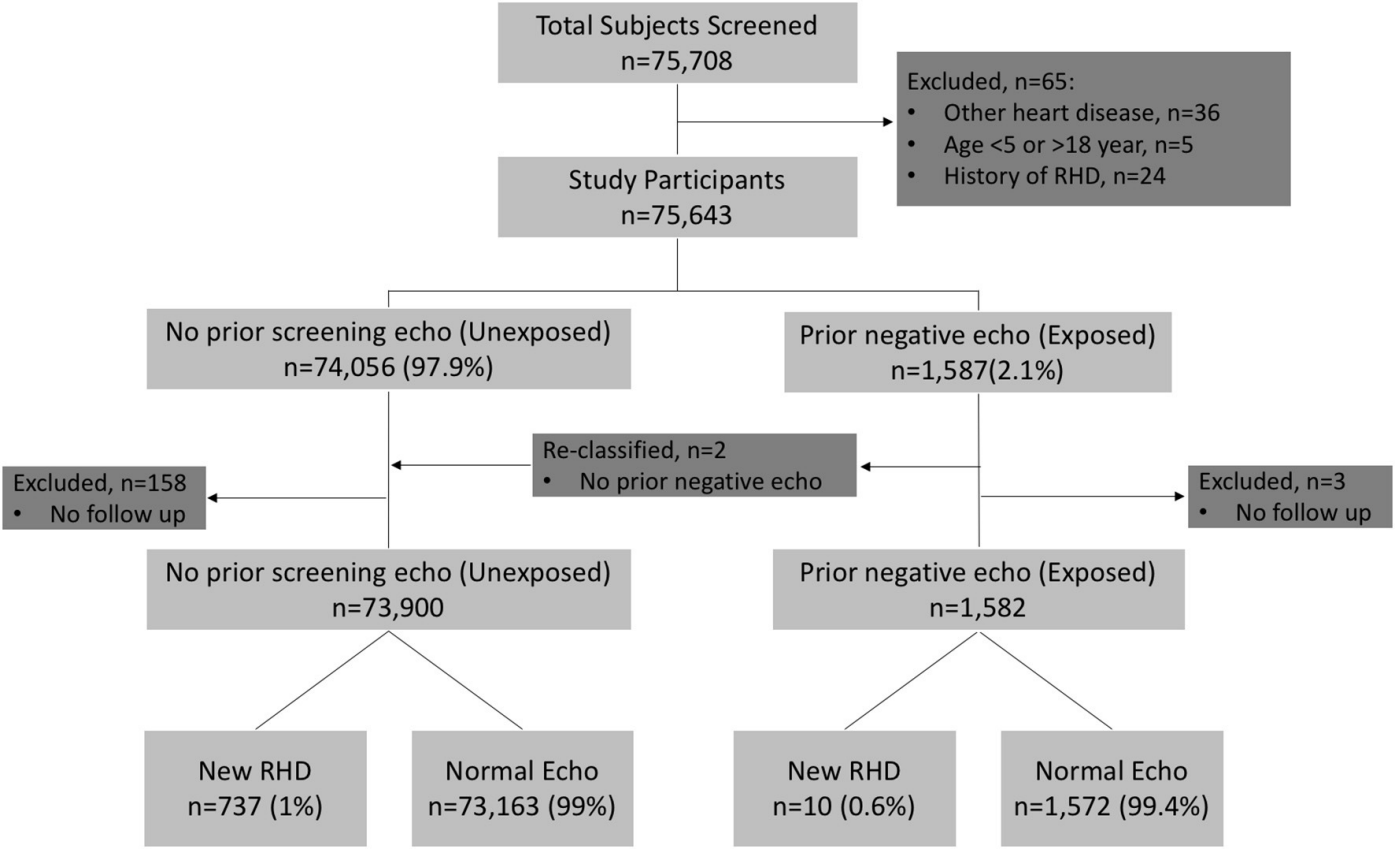
Flow Diagram for Study Participants.

A total of 75,708 children underwent echo screening during the second echo screening period. Sixty-five children were excluded from the study: 36 were diagnosed with another form of heart disease (32 with congenital heart disease; 4 with cardiomyopathy), 5 were outside the study age range (<5 years or >18 years of age), and 24 had been previously diagnosed with RHD before the time of the initial screening echo (Figure 1). A total of 75,643 subjects were initially included, 1,587 (2.1%) of whom had a previous negative screening echo, making up the exposed cohort, and 74,056 (97.9%) with no prior screening echo, making up the unexposed cohort. Two subjects were reclassified from exposed to the unexposed cohort, as they had been clinically diagnosed with RHD at the Gulu Regional Referral Hospital (GRRH) in between the initial and second screening periods and had not in fact undergone an initial screening echo as part of initial screening studies. An additional 3 (0.1%) subjects were excluded from the exposed cohort, and 158 (0.2%) were excluded from the unexposed cohort for not returning for confirmatory echo (Figure 1). This left 1,582 subjects in the exposed cohort and 73,900 in the unexposed group. In the exposed cohort, the median age was 13 years (IQR 10–16) and 693 (43%) were male. Time from the initial echo to the second screening echo ranged from 44 to 60 months (3.6–5 years). To confirm adequacy of recall, a subset (33%) of the exposed cohort data was crosschecked with prior records and found to have accurate recall.

In the exposed cohort, there were 10 new cases of RHD; a prevalence of 0.6% (10/1,582). There were 737 new cases of RHD in the unexposed cohort; a prevalence of 1% (737/73,900). The relative risk of developing RHD if a child had a prior negative screening echo compared to those with no prior screening echo was determined to be 0.64 (95% CI 0.3 to 1.2, *p* = 0.15). The relative risk reduction was 0.4, or a 40% reduced risk of newly diagnosed RHD in those who had a previously negative screening echo compared to those who had never been screened before. There was no difference in age or gender between those who developed RHD and those without RHD in the exposed cohort (Table 1) or when comparing the RHD positive cases in the exposed and unexposed cohorts (Table 2). Of the 10 new cases of RHD in the exposed cohort, 9 (90%) were borderline, 1 (10%) was mild definite RHD, and none were moderate or severe. Of the 737 new cases of RHD in the unexposed cohort, 566 (76.8%) were borderline, 152 (20%) were mild definite, and 19 (2.6%) were moderate or severe RHD (Table 2).

**Table 1:** Demographic data of children with a prior negative screening echo (exposed cohort) with and without RHD.

	**Exposed with RHD (*n* = 10)**	**Exposed without RHD (*n* = 1,572)**	^*^p-value
**Age (years), mean (std dev)**	14.3 (1.2)	13.5 (1.8)	0.199
**Gender**			
Male (%)	4 (40%)	678 (43%)	0.841
Female (%)	6 (60%)	893 (67%)	

* *To assess the differences between participants in the exposed cohort with RHD vs. without RHD, p-values were calculated using chi-square tests for categorical variables and independent t-tests for continuous variables*.

*RHD, Rheumatic heart disease; Std dev, Standard deviation*.

**Table 2:** Demographic data of children with new RHD, exposed and unexposed.

	**RHD in exposed (*n* = 10)**	**RHD in unexposed (*n* = 737)**	^*^p-value
**Age (years), mean (std dev)**	14.3 (1.2)	12.6 (2.9)	0.069
**Gender**			
Male (%)	4 (40%)	318 (43%)	0.883
Female (%)	6 (60%)	419 (57%)	
**Severity of RHD**			
Borderline/mild (%)	10 (100%)	718 (97.4%)	0.606
Moderate/severe (%)	0 (0%)	19 (2.6%)	

* *To assess the differences between participants in the exposed cohort with RHD vs. without RHD, p-values were calculated using chi-square tests for categorical variables and independent t-tests for continuous variables*.

*RHD, Rheumatic heart disease; Std dev, Standard deviation*.

## Discussion

This is the first population-based study to examine the risk of developing RHD over time in those with a previously normal screening echo. Our data demonstrate no statically significant difference in rates of RHD development between those with a previously negative screening echo and those never screened before. However, there was a trend demonstrating lower rates of RHD and less severe RHD in those with a prior negative screening echo, and a 40% risk reduction of detecting RHD on a follow up screening echo, when a child had a negative screening echo in the past 3–5 years. While not powered to study severity of disease, this study found that children with a previously negative screening echo did not develop advanced RHD.

Three prior studies also report on the development of RHD in children with a previously normal echocardiogram. An Australian study evaluated the progression of minor echocardiographic changes over time. The study included a comparison group of 325 children with initially normal echos and found that 19 (5.9%) had evidence of RHD at an average follow up of 3.5 years (9). A second study out of South-Pacific New Caledonia followed a cohort 114 children with RHD, diagnosed by echo screening and 227 healthy controls (8). After a median follow-up period of 2.58 years, 31 (13%) of the healthy controls had evidence of RHD on echo screening, where only 2 were definite RHD and 29 were borderline RHD (8). Both studies demonstrate very high rates of RHD development in children who once had a normal echocardiogram, even higher than baseline population prevalence. It would require further study to fully understand such high rates of conversion from normal to abnormal echo findings in these populations.

In a third study out of Fiji, Engelman et al. reported clinical outcomes for 70 screen-negative cases with a median of 7.4 years and found no deaths or RHD-related admissions (7). Albeit in a small cohort size, this demonstrates that over a longer period of time, complications related to RHD may be rare in those with a previously negative screening echo. Similarly, our study found no advanced disease among those who had a previously negative screening echo. While our study was not designed to show differences in disease severity, the results, along with Engelman's study, do suggest that children with prior negative screen are unlikely to develop severe disease over a 3–7 year timeframe. This can help inform future research efforts and planning for currently existing screening programs.

The overall prevalence of RHD in children screened in this study was only 1%, which was lower than anticipated. Previous screening studies in this region have shown a prevalence of 2.5–3% (13, 17, 18). While this study was not designed to understand this difference, it is possible that multiple prior echo screening studies, which included RHD education in schools, health centers, and in the community, may have increased awareness and subsequently reduced community rates of new RHD through improved primary and secondary prevention. The lower overall prevalence in both cohorts in this study may have affected the ability to detect a significant difference between the cohorts. This study was designed with a conservative baseline RHD population estimate of 2%, and hypothesized a 50% risk reduction between the cohorts. If the baseline prevalence in our study had been 2%, the findings would have been statistically significant, even if the prevalence of the exposed cohort increased up to 1%. For the cohort who underwent a prior screening echo, there were no ongoing health campaigns or programs specifically targeting this population that would have led to a significant change in the risk of developing RHD overtime. In addition, multiple studies in this region have continued to show poor health-seeking behavior in children and adults with symptoms of acute rheumatic fever (19, 20). Therefore, it is unlikely that community intervention or education altered the rates of RHD in those with a previously negative screening echo any more that in those without a prior screening echo.

The pragmatic design of this study led to multiple limitations. Most importantly, our primary endpoint was underpowered as a result of finding fewer than anticipated children who had prior echo screening, and due to a lower baseline RHD prevalence in newly screened children. Second, this study relied on recall from children to determine if they had a previous screening echo 3–5 years prior. As is true in most developing regions of the world, children in Northern Uganda have limited, or no, health records, medical record numbers, or social security numbers. In addition, their birth dates and name spelling are frequently inconsistent and unreliable. As a result, recall was used in lieu of more reliable data. Cross-checking one-third of the exposed sample found recall to be accurate, but we were unable to cross-check for false-negative reporting. Finally, the echo screening was performed by healthcare professionals with varying levels of ultrasound experience, including sonographers, cardiologists, and cardiology fellows from both the U.S. and Uganda. However, the same staff was used throughout screening, so missed cases of RHD by inexperienced staff would have led to non-differential misclassification. In effect, this may have skewed the results toward the null hypothesis: no difference in risk between the cohorts.

Despite these limitations, this study adds important and novel information to the field of echocardiographic screening for the diagnosis of RHD, at a time when the use of echo screening is being debated as a universal measure in endemic settings. The RHD community lists secondary prevention as one of the four crucial pillars to decrease the burden of RHD, as described by the late Mayosi (21), but this cannot be accomplished without identification of disease. Echocardiographic screening is known to detect subclinical disease, but further research is needed to support determine the proper use and implementation in endemic regions. Studies such as this one are critically examining the use of echo screening, so that it can be properly utilized as a vital tool in the domain of secondary prevention. This data provides valuable information about intervals of echo screening over time, as well as the lack of disease progression, and lack of disease severity, in those with previously negative screening echos. RHD largely remains a disease of the poor and disadvantaged, and therefore, resource allocation is vital in these communities. Further research in this realm will help distinguish proper screening intervals, appropriate age of screening, and how best to screen large populations to efficiently and economically incorporate screening into RHD endemic regions of the world to effectively reduce the burden of disease.

## Conclusion

Our study found no statistical difference in RHD prevalence between children with previously normal echocardiograms and children who had never been screened, however, there was a trend toward decreased risk. These data call for a large-scale controlled cohort study to evaluate the risk of developing RHD after a negative screen, powered to look at differences in age at first negative screen, the interval between screenings, and the severity of disease. This information has important implications for the design of population screening programs and the use of echocardiographic screening in endemic RHD regions.

## Data Availability Statement

The raw data supporting the conclusions of this article will be made available by the authors, without undue reservation.

## Ethics Statement

The studies involving human participants were reviewed and approved by Institutional Review Board (IRB), Mulago Hospital at Makerere University, as part of the National RHD Outreach Program (REC REF 2013-072). Written informed consent for participation was not provided by the participants' legal guardians/next of kin because: Echocardiographic screening was performed under the national RHD Outreach Program.

## Author Contributions

MZ, CS, and AB prepared the written manuscript. MZ, AS, AD, J-LN, IO, AT, JR, EO, CS, and AB reviewed and edited the manuscript. AS, AD, J-LN, IO, JR, and EO contributed to data gathering. AS, AD, and AT contributed to data organization. MZ, CS, and AB contributed to data analysis. All authors contributed to the article and approved the submitted version.

## Conflict of Interest

The authors declare that the research was conducted in the absence of any commercial or financial relationships that could be construed as a potential conflict of interest.
